# Supplementary material for: Non-antibiotics disrupt colonization resistance against enteropathogens
Source: Nature. 2025 Jul 16;644(8076):497–505. doi: 10.1038/s41586-025-09217-2 (PMC12350171; doi:10.1038/s41586-025-09217-2)
Supplement: Supplementary file 1 — Supplementary Note, Supplementary Fig. 1 and a guide to Supplementary Tables 1–14. [file 41586_2025_9217_MOESM1_ESM.pdf]

nature portfolio

https://doi.org/10.1038/s41586-025-09217-2

Supplementary information

Non-antibiotics disrupt colonization resistance against enteropathogens

In the format provided by the authors and unedited

Supplementary Text

Direct pathogen-commensal interactions are poor predictors of *S*. Tm growth in Com20

Since drug exposure led to compositional shifts in Com20 that were linked to *S*. Tm expansion, we examined which species drove changes in the community structure in each of the colonization groups. Compared to untreated controls, communities resulting from *S.* Tm-favoring treatments were enriched in *Collinsella aerofaciens*, *Enterocloster bolteae*, *Dorea formicigenerans*, and *Agathobacter rectalis* and were depleted in *Phocaeicola vulgatus*, *Bacteroides fragilis*, *Bacteroides uniformis*, and *Streptococcus parasanguinis* (**ED Fig. 6d**). Similar species drove the differences between controls and the *S*. Tm-neutral group, albeit with smaller effect sizes (**ED Fig. 6d**), while *Sarcina perfringens* (also known as *Clostridium perfringens*), *Veillonella parvula*, and *Fusobacterium nucleatum* were depleted in the *S*. Tm-restricting group (adjusted *P* values < 0.1 in all cases) (**Fig. 2c, ED Fig. 6d**).

We evaluated the interaction between *S*. Tm and individual members of the community using two approaches. First, we cultured *S*. Tm with each commensal independently. Contrary to our expectations based on the enrichment patterns observed in the drug-treated communities, multiple species, including *E. bolteae* and *C. aerofaciens,* significantly inhibited *S*. Tm growth (two-tailed t-test. adj. *P* value < 0.1 in both cases. **ED Fig. 6h**). No species resulted in an increased growth of the pathogen.

Next, we tested whether dropout of individual members of Com20 could result in changes in community composition and pathogen growth in the absence of drug treatment. The absence of most members resulted in small changes in the composition of the microbial community, with the exception of *S. perfringens* dropout (**ED Fig. 6e-g**). Intriguingly, *S. perfringens* had a high relative abundance in untreated Com20 (mean abundance ± SD: 43.4 % ± 6.7. **Fig. 1b**) and pairwise culture with *S*. Tm resulted in a significantly lower pathogen growth **(ED Fig. 6h;** *P* values: *S. parasanguinis* 0.037, *F. nucleatum* 0.036, *E. ramosa* <0.001, *R. gnavus* 0.003, *S. saccharolytica* <0.001, *S. perfringens* 0.003, *E. lenta* 0.009, *C. aerofaciens* < 0.001, *E. boltea* < 0.001; two-tailed t-test), although this species was depleted in *S*. Tm-restricting communities compared to controls (**ED Fig. 6d**). Similarly, growth of the pathogen was lower in communities without *Parabacteroides merdae* and *E. lenta,*

while it was higher in those lacking *B. uniformis*, *Bariatricus comes*, *Roseburia intestinalis* and *A. rectalis* (adj. *P* value < 0.1 in all cases).

Our observations suggest that *S*. Tm invasion is a complex phenomenon that cannot be fully predicted from pairwise interactions between the pathogen and individual commensals. Moreover, the absence of a particular competitor can result in a different community state that restricts *S*. Tm growth, further highlighting the importance of the ecological context for pathogen-commensal interactions.

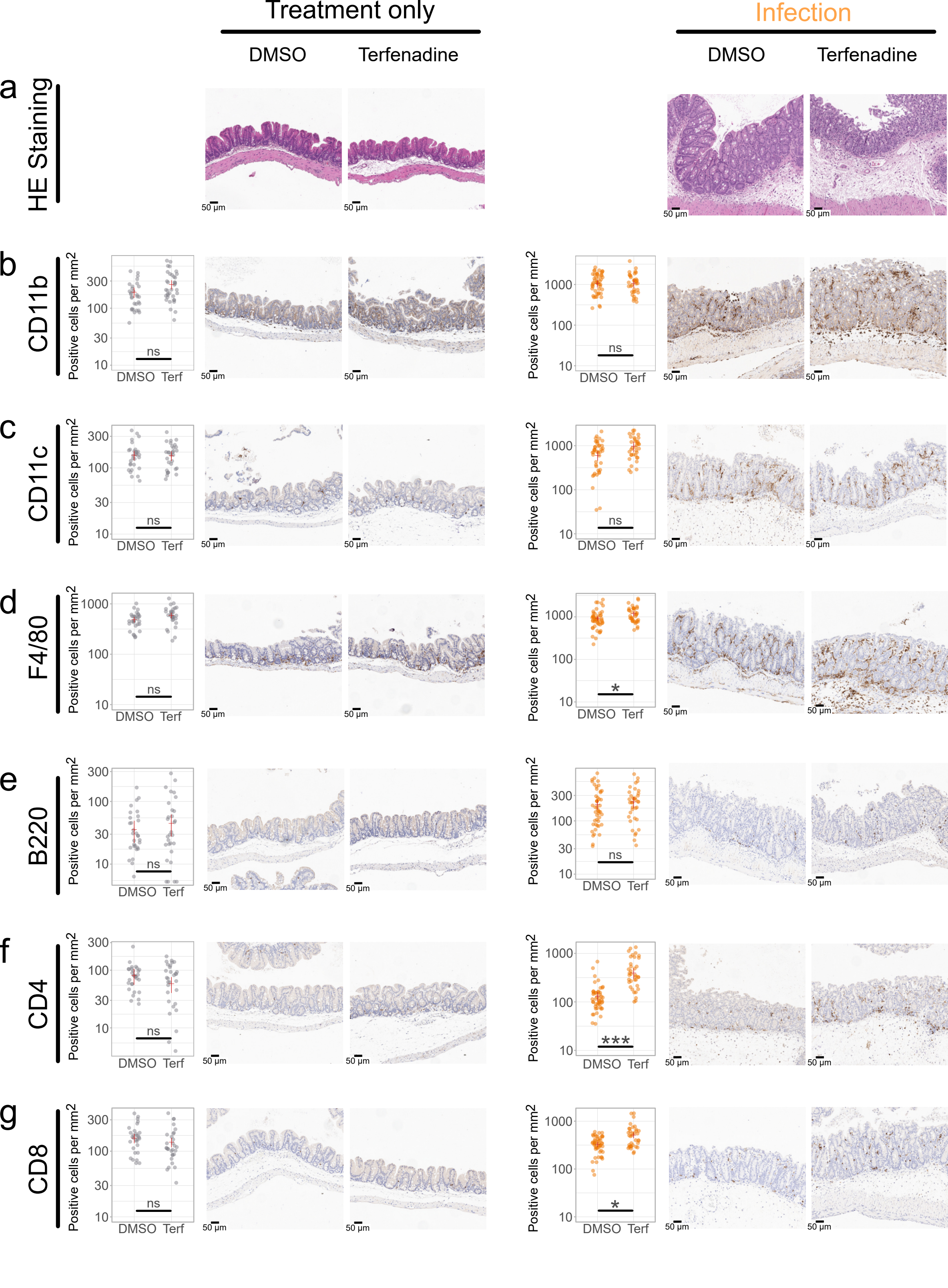

To be continued on subsequent page

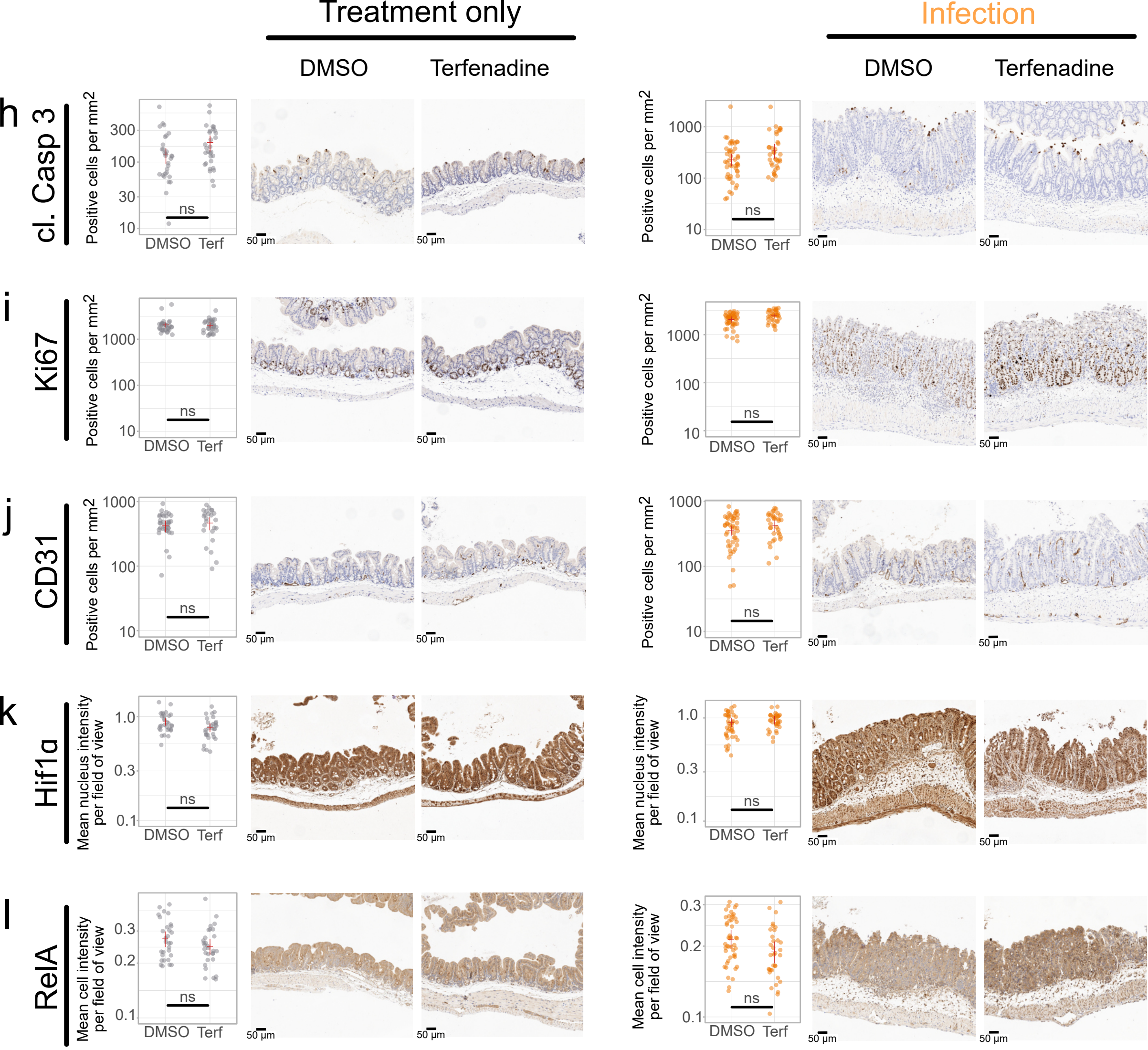

Supplementary Figure 1: Terfenadine treatment results in increased mucosal inflammation 4 days after infection but not prior to infection. Representative photomicrographs of cecal sections of humanized mice infected with *S*. Tm (orange) and non-infected controls (gray) after **a**) hematoxylin and eosin (HE) and **b-l)** immunohistochemical stainings for key cellular processes, including immune cell infiltration (CD11b (b), CD11c (c), F4/80 (d), B220 (e), CD4 (f), CD8 (g)), apoptosis (cleaved caspase-3 (h)), proliferation (Ki67(i)), endothelial function (CD31 (j)), hypoxic adaptation (Hif1α (k)), and inflammation (RelA (l)). Stainings were quantified using a pathoscore for *S*. Tm infection (HE), the number of positive cells per mm² (CD11b, CD11c, F4/80, B220, cleaved caspase-3, Ki67, CD31), mean nuclear staining intensity (Hif1α), or mean cellular staining intensity (RelA). Dots represent values for six non-independent fields of view within each mouse. The hierarchical nature of the data, along with the non-independence of observations, was explicitly addressed by fitting generalized linear mixed models. Red horizontal and vertical lines depict the resulting marginal mean estimates with their associated standard errors. Contrasts were evaluated using two-sided Wald z-tests. *P* values for group contrasts from the regression model are shown. ns: not significant; $p > 0.05$.

Supplementary tables

Supplementary Table 1

Prestwick library screen results for pathogenic *Gammaproteobacteria* species, including *H. parainfluenzae*, *S. enterica* serovar Typhimurium, *S. flexneri*, *Y. pseudotuberculosis*, and *V. cholerae.*

Supplementary Table 2

IC25 values for 65 drugs used to treat 5 pathogens and 19 gut commensal species.

Supplementary Table 3

Prestwick library screen in *S*. Tm WT, *S*. Tm Δ*tolC*, *P. vulgatus* WT, *P. vulgatus* ΔBVU_1672-1675.

Supplementary Table 4

Results of the *S*. Tm *in vitro* challenge assay in Com20.

Supplementary Table 5

Results of the *in vitro* challenge assay for additional pathogenic *Gammaproteobacteria*.

Supplementary Table 6

KEGG pathways overrepresented in the set of differentially expressed genes in *S*. Tm in pure culture or after invasion of Com20 treated with terfenadine, clomiphene, floxuridine, simvastatin compared to untreated Com20.

Supplementary Table 7

KEGG pathways overrepresented in the top 20 % of genes with the highest expression on each member of Com20 after treatment of the community with floxuridine, simvastatin, or in the untreated community.

Supplementary Table 8

Results of the *S*. Tm *in vitro* challenge assay in Com21.

Supplementary Table 9

Log2-fold change in the luminescence signal of *S*. Tm in stool-derived communities after drug treatment.

Supplementary Table 10

Differential abundance of bacterial taxa in Com20-colonized drug-treated mice compared to untreated mice.

Supplementary Table 11

Differential abundance of bacterial taxa in drug-treated SPF mice compared to untreated mice.

Supplementary Table 12

Differential abundance of bacterial taxa in drug-treated Humanized mice compared to untreated mice.

Supplementary Table 13

Key resources used in this study.

Supplementary Table 14

Accession ID of genomes used in this study.
